# Genome wide analysis of kinesin gene family in *Citrullus lanatus* reveals an essential role in early fruit development

**DOI:** 10.1186/s12870-021-02988-6

**Published:** 2021-05-10

**Authors:** Shujuan Tian, Jiao Jiang, Guo-qi Xu, Tan Wang, Qiyan Liu, Xiner Chen, Man Liu, Li Yuan

**Affiliations:** State Key Laboratory of Crop Stress Biology for Arid Areas, College of Horticulture, Northwest A&F University, Yangling, 712100 Shaanxi China

**Keywords:** Kinesin genes, Expression patterns, Early fruit development, Hormones response, *Citrullus lanatus*

## Abstract

**Background:**

Kinesin (KIN) as a motor protein is a versatile nano-machine and involved in diverse essential processes in plant growth and development. However, the kinesin gene family has not been identified in watermelon, a valued and nutritious fruit, and yet their functions have not been characterized. Especially, their involvement in early fruit development, which directly determines the size, shape, yield and quality of the watermelon fruit, remains unclear.

**Results:**

In this study, we performed a whole-genome investigation and comprehensive analysis of kinesin genes in *C. lanatus*. In total, 48 kinesins were identified and categorized into 10 kinesin subfamilies groups based on phylogenetic analysis. Their uneven distribution on 11 chromosomes was revealed by distribution analysis. Conserved motif analysis showed that the ATP-binding motif of kinesins was conserved within all subfamilies, but not the microtubule-binding motif. 10 segmental duplication pairs genes were detected by the syntenic and phylogenetic approaches, which showed the expansion of the kinesin gene family in *C. lanatus* genome during evolution. Moreover, 5 *ClKINs* genes are specifically and abundantly expressed in early fruit developmental stages according to comprehensive expression profile analysis, implying their critical regulatory roles during early fruit development. Our data also demonstrated that the majority of kinesin genes were responsive to plant hormones, revealing their potential involvement in the signaling pathways of plant hormones.

**Conclusions:**

Kinesin gene family in watermelon was comprehensively analyzed in this study, which establishes a foundation for further functional investigation of *C. lanatus* kinesin genes and provides novel insights into their biological functions. In addition, these results also provide useful information for understanding the relationship between plant hormone and kinesin genes in *C. lanatus*.

**Supplementary Information:**

The online version contains supplementary material available at 10.1186/s12870-021-02988-6.

## Background

Kinesins, widely distributed in all eukaryotic organisms [1], are a group of microtubule-based motor proteins that move along microtubule (MT) protofilaments, powered by hydrolyzing ATP, to drive various essential biological processes [2]. All kinesin proteins share a conserved motor domain of approximately 350 amino acids. The “motor head” domain consists of an ATPase catalytic site and MT-binding sites, which possesses catalytic ATPase and MT-binding abilities [3]. The kinesin family is classified as N-type, the middle and C- type kinesins respectively, with the motor head domain at or near the N-terminus, in the middle, and close to the C-terminus of the molecule. The “motor head” domain is followed by the stalk region and the “small globular tail” at the opposite end of the kinesin molecule. The “motor head” domain is responsible for protein movement powered by ATP hydrolysis [4–6]. And the “stalk/tail” domain is important for the interaction with subunits of the holoenzyme or with the cargo molecules [4, 7, 8]. The short “neck” region between the “head” and “stalk/tail” is essential for functions such as the direction of motility or regulation of activity [9]. The motor domain is well conserved in each kinesin subfamily, whereas the stalk/tail region outside the motor domain is highly divergent even in the same subfamily, reflecting the diverse biological functions even within the same subfamily.

Based on phylogenetic analysis using the conserved motor domain sequences, the kinesins are divided into fourteen families, designated as kinesin-1 to kinesin-14. Kinesins that do not belong to any of these subfamilies are considered as orphans, but most kinesins identified can easily be assigned to a specific family [10]. Most members of kinesin families have an N-terminal motor domain named as N-type kinesins whereas few families have an internal motor domain or a C-terminal motor domain. The directionality of kinesins varies between families, which is sometimes correlated with the position of the motor domain. In general, kinesins with the N-terminal motor domain travel to the plus ends of MTs whereas the C-terminal motors move toward the minus ends of MTs [11–14].

Previous expression profiles analysis revealed that plant kinesin genes play important roles in fruit development. In apple (*Malus domestica* Borkh.) cultivar Fuji, the kinesin gene *KIN2* was strongly expressed in early stage of fruit development [15]. Further investigation showed that *KIN2* gene was also expressed primarily in two other apple genotypes “Gala” and “Golden Delicious” [16]. This demonstrated that the kinesin gene *KIN2* carries out regulatory role in early fruit development in apple. In cucumber, the kinesin genes *CsKF1-7* were highly expressed during early fruit development and involved in rapid cell division or expansion [17]. Moreover, the *CsKF1* and *CsKF3* were dramatically active in the fruit elongation stages, implicating their essential roles in the fruit length regulation in cucumber [18]. Intriguingly, in tomato (*Solanum lycopersicum*), the kinesin gene *SpPAKRP* was predominantly expressed in the placenta tissue in the 4-DPA (Days Post Anthesis) fruit, so *SpPAKRP* is likely involved in controlling early fruit development by regulating placenta development [19, 20]. The watermelon fruit as well as tomato is classified as a berry fruit and the edible parts of the fruit develop from placenta [21]. In addition, the size, yield and quality of the cucurbits fruit depend on the regulation of the placenta during early fruit development [22–24]. So, identification and the functional analysis of kinesin is the ideal entry point for exploring molecular mechanism of kinesin genes in regulating watermelon early fruit development. Moreover, other kinesin genes have been verified to participate in different biological functions, including root, stem and leaf various vegetative tissues development genes (e.g., *DBS1*, *BC12/GDD1*, *AtKINESIN-4A/FRA1*) [25–28]; plus anther, male gametophyte, embryo, endosperm and seed development genes ( e.g., *SRS3* and *NtKRP*) [29–31]. These works revealed the critical roles of plant kinesins in many essential processes in plant development, including not only plant vegetative growth but also plant reproductive process. However, up to now, little is known about kinesin family genes in watermelon. And very few kinesins have been functionally identified during early fruit development. Therefore, it is well worthy to extensively investigate their roles in economic crops, like watermelon.

Watermelon is the fifth consumed fresh fruit in the world. It is a highly nutritional valued fruit, known as “the king of summer fruits”. The early fruit development directly determines the size, shape, yield and quality of the watermelon fruit. Considering the involvement of kinesin genes in early fruit development via regulating placenta development in tomato [19, 20], genome-wide study of the kinesin genes in watermelon is the ideal pointcut for exploring their real roles in the critical developmental processes, especially in watermelon early fruit development. Thus, kinesin genes were analyzed in the *Citrullus lanatus* genome in this study. The phylogenetic relationships, gene structure, chromosomal locations, and conserved motifs of the encoded proteins were also investigated. The tissue-specific expression patterns of all *ClKINs* genes in watermelon were further studied, as well as *ClKINs* expression under hormone-treated condition. Particularly, five *ClKINs* genes showed specific and abundant expression in early fruit development stage. Our work provides useful information regarding the molecular mechanism of kinesin genes regulating early fruit development and a new insight into the yield and quality control mechanism in watermelon.

## Results

### Genome-wide identification of kinesin genes in *Citrullus lanatus*

A total of 63 candidate genes were identified from the watermelon genome (Cucurbit Genomics Database, http://www.icugi.org/) based on amino acids sequence analysis. 15 candidate genes didn’t contain conserved kinesin motor domain and then were excluded from further analysis. In addition, these remaining 48 Kinesin genes can also be verified by hidden Markov models (HMMs) analysis search of function conserved Pfam domains, which was consistent with the above sequence similarity blasting. In conclusion, 48 kinesin genes with complete and functional structures are presented in the watermelon genome, designated as *ClKINs* hereafter.

To explore basic properties of each kinesin, the lengths of genome DNA and protein sequences, the numbers of the introns and exons, the isoelectric point and the theoretical molecular weight were predicated, respectively (Table S1). The kinesin genes in *Citrullus lanatus* genome have coding sequence lengths of 927-8670 base nucleotides, encoding proteins length ranged from 308 to 2889 amino acids with predicted molecular weight in the range of 25.0-330.3 KDa. The theoretical isoelectric point calculation indicated that the kinesin protein isoelectric points (pI) were distributed in the range of 5.10-9.65 (Table S1).

**Fig. 1:**
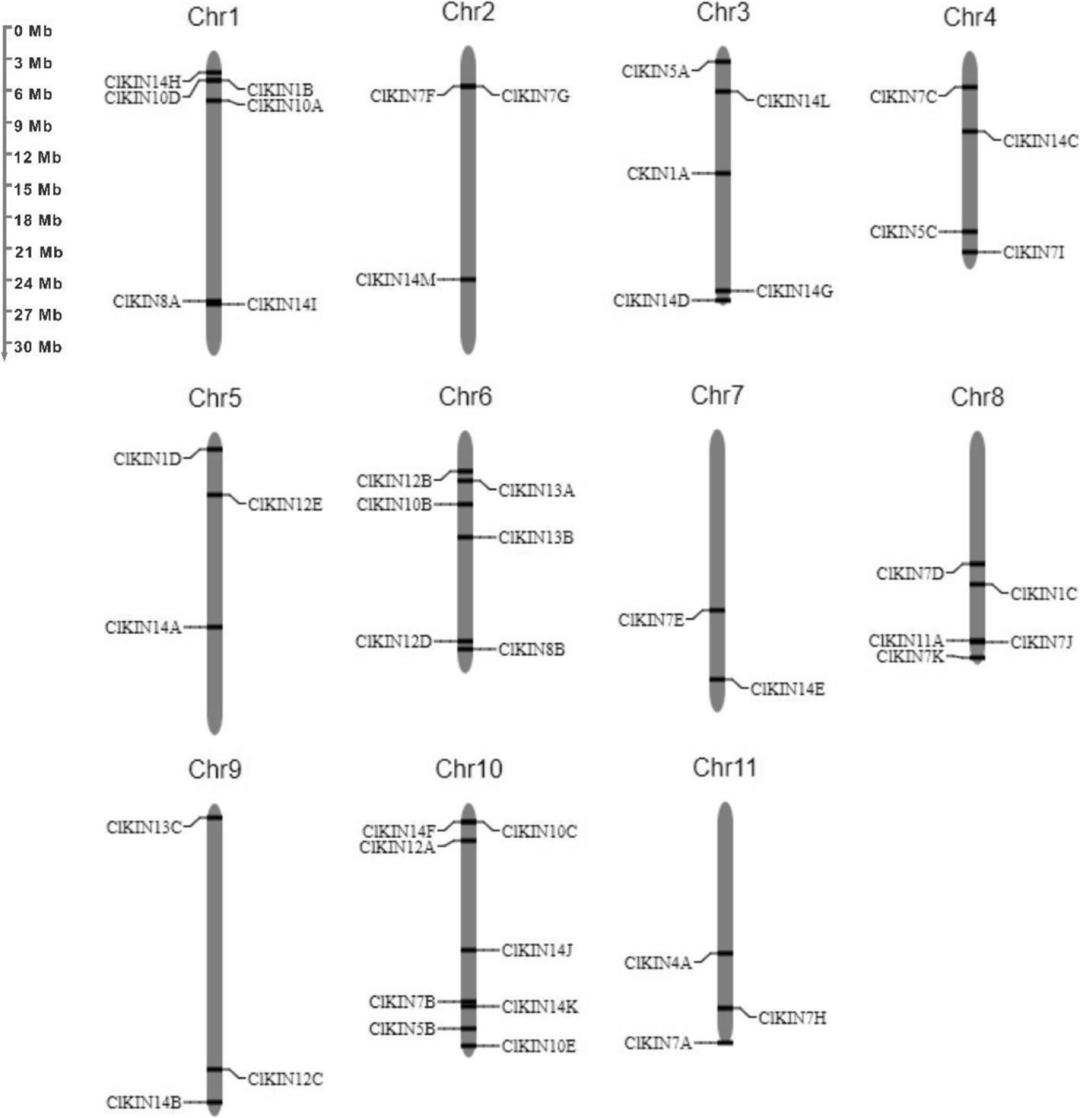
Distribution of *Citrullus lanatus* kinesin genes on 11 chromosomes. Chromosome numbers were marked as Chr1- Chr11 at the top of each chromosome. The sizes of chromosome were labeled on the left of the figure. Forty-eight kinesin genes of watermelon were mapped to different chromosomes using Map Chart

In order to characterize the distribution of kinesin genes in the watermelon genome, the physical locations of kinesin genes on the watermelon chromosomes were further investigated. 48 kinesin genes were mapped to the 11 chromosomes (Fig. 1), exhibiting an uneven distribution in the watermelon genome. Chr10 contains the maximum numbers of 8 kinesin genes, while only two genes locate on Chr7. The other chromosomes, including Chr1, Chr2, Chr3, Chr4, Chr5, Chr6, Chr8, Chr9, Chr11, contain 3-6 kinesin genes, respectively.

### Phylogenetic analysis of kinesin family

**Fig. 2:**
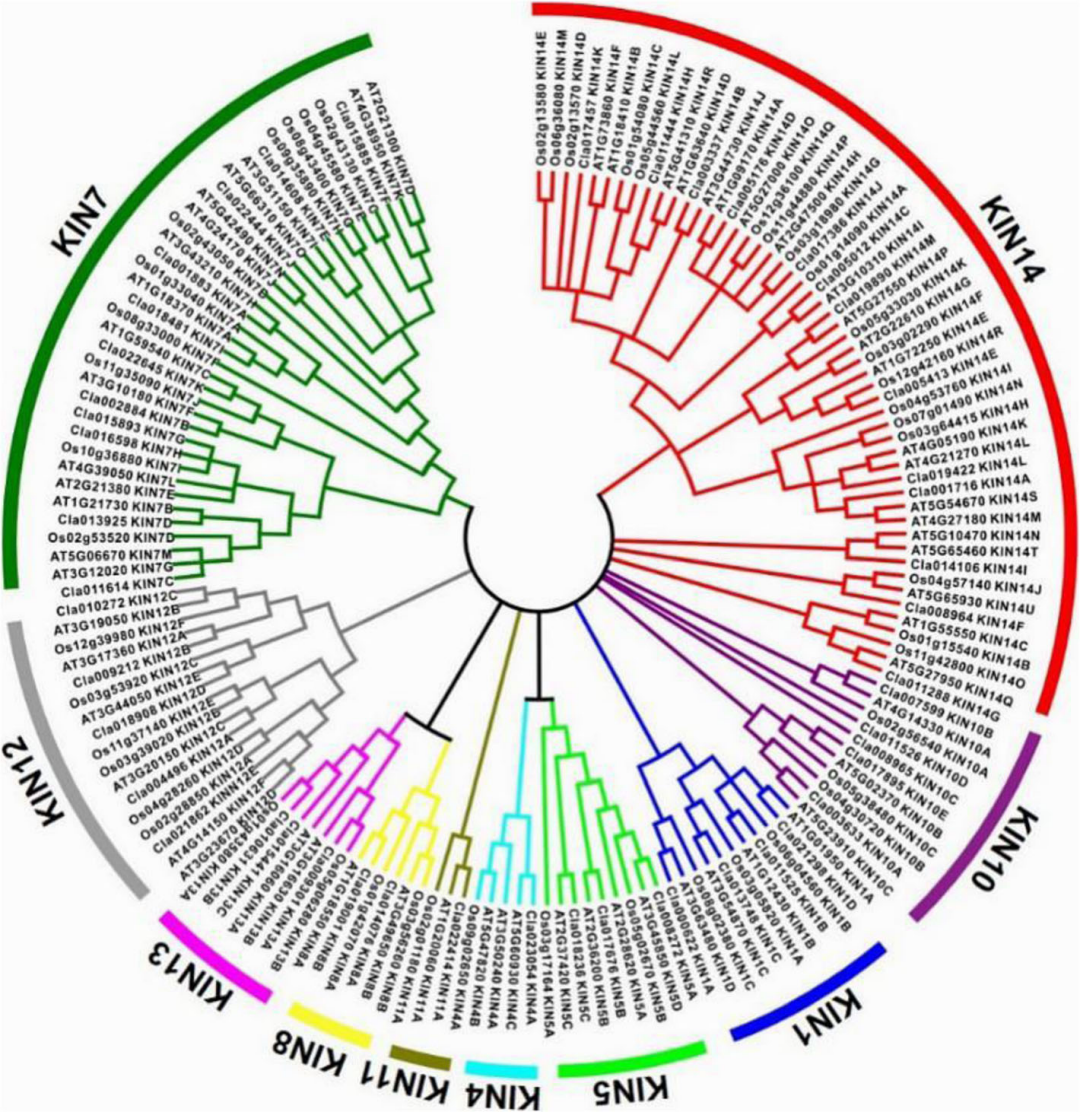
Phylogenetic relations of the kinesins from *C. lanatus*, *A. thaliana* and *O. sativa*. The tree was calculated with MEGA6.06 software using neighbor-joining method. Neighbor joining phylogenetic tree of the kinesin family. This tree summarizes the evolutionary relationships among kinesins in watermelon based on the kinesin sequences from Arabidopsis and rice. The length of the branches is proportional to the amino acid variation rate. Watermelon possess 10 kinesin families, including kinesin-1,4, 5, 7, 8, 10, 11, 12, 13, 14 families. In addition, the kinesin-7 and kinesin-14 families are greatly expanded

To estimate the phylogenetic relationships of watermelon kinesins to other known kinesins in different plants, multiple sequence alignment of watermelon kinesins motor domain sequences to the sequences from dicotyledonous model plant, *A. thaliana,* and monocotyledonous crop, *O. sativa*, was conducted using the software MUSCLE and the phylogenetic tree of these kinesins was then generated with MEGA 6.06 using neighbor joining method (Fig. 2). The phylogenetic analysis suggested that kinesin proteins from three different species can be categorized into 10 families: KIN1, KIN4, KIN5, KIN7, KIN8, KIN10, KIN11, KIN12, KIN13, KIN14 (Figure S1 and Figure S2). Among them, the KIN14 family is the largest family consisting of 52 kinesins. The KIN7 family is the second largest family, which has 36 kinesin members. KIN11 is the smallest family with only 3 kinesins. Kinesin numbers from watermelon followed the same distribution tendency as the other two species. 13 watermelon kinesins belong to the KIN14 subfamily which is the largest family of all. 7 kinesins are grouped into KIN7, the second largest subfamily. Only 1 kinesin is in KIN11 family, representing the smallest subfamily. To further explore the evolutionary relationships among kinesin in fruit plants, we identified 53 kinesin homologs from fruit plant tomato. The phylogenetic analysis showed that the kinesin-14 and 7 subfamilies were expanded during evolution, which is consistent with Arabidopsis and rice (Figure S3). The phylogenetic relationship does not show any recognizable distinction between dicot and monocot species analyzed, indicating functional conservation of kinesin throughout plant kingdom.

### Gene structure and conserved motif distribution analysis of watermelon kinesin family genes

**Fig. 3:**
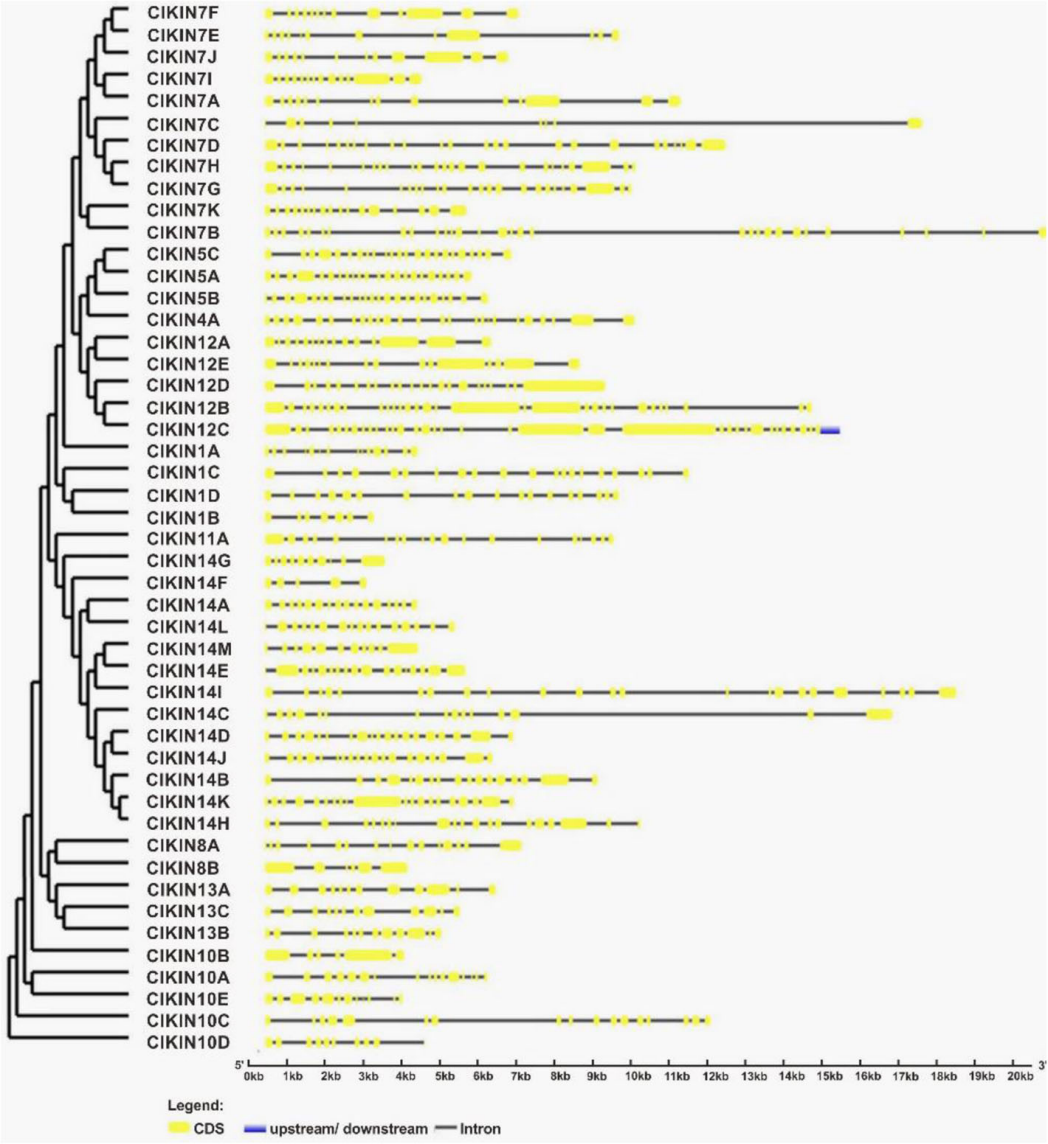
Genomic structures of kinesin genes in *C. lanatus*. The *ClKINs* gene structures were visualized by using the program GSDS2.0 software. The gene structures and intron/exon arrangements of the *ClKINs* genes were determined by the comparison of the cDNA sequence of each *ClKIN* with its genomic DNA sequence. The intron number of all watermelon *ClKINs* genes ranged from 4 to 34. *ClKIN14F* only has 4 introns while there are 34 introns presented in *ClKIN12C*

The gene structure and intron/exon arrangements of the *ClKINs* genes were determined by the comparison of the cDNA sequence of each *ClKIN* with its genomic DNA sequence. The analysis results revealed that the intron number of all *ClKINs* genes ranged from 4 to 34. *ClKIN14F* only has 4 introns while there are 34 introns presented in *ClKIN12C* (Fig. 3).

**Fig. 4:**
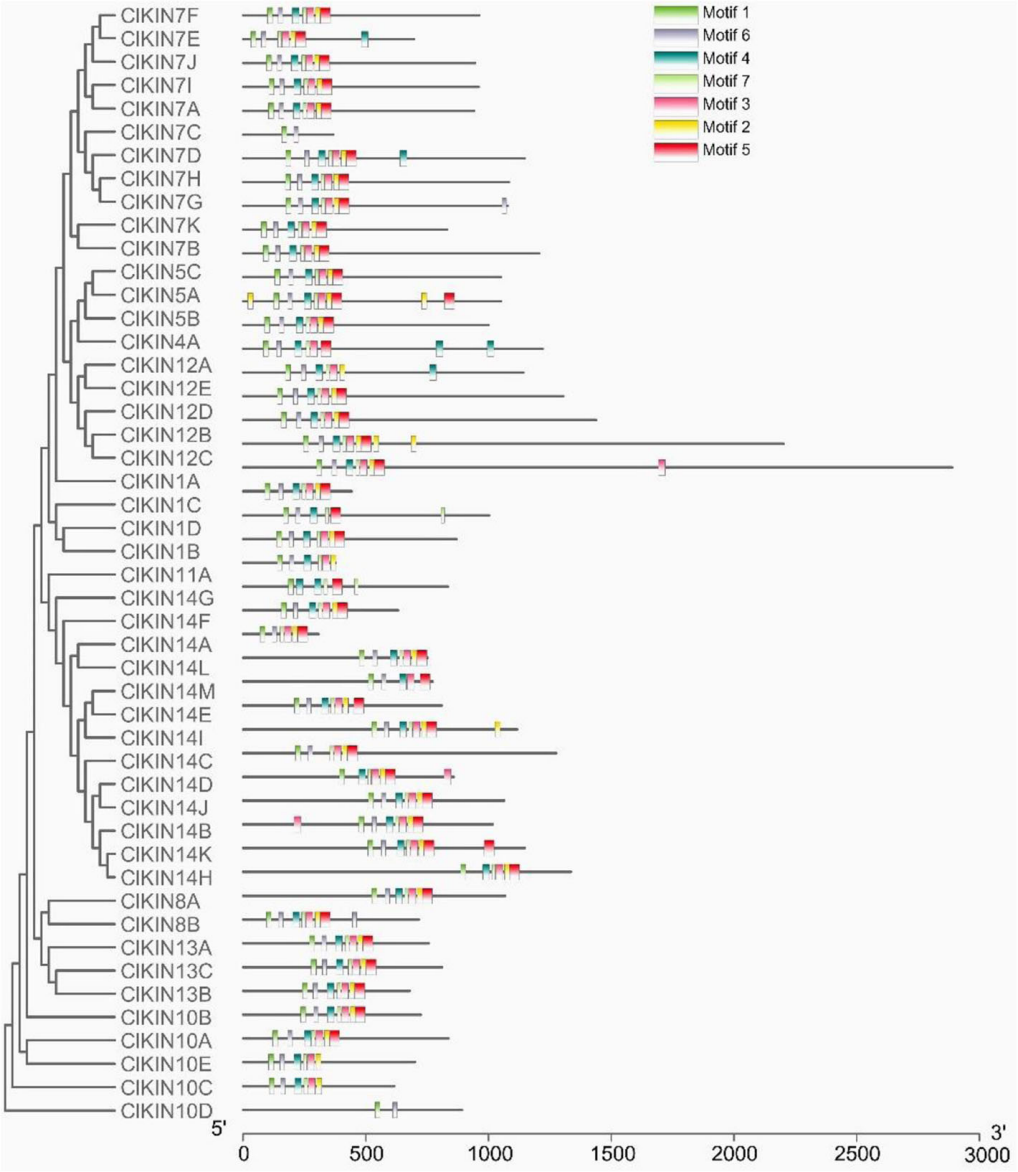
Distributions of conserved motifs in watermelon kinesin proteins. The phylogenetic tree of *ClKINs* is on the left panel. The motifs of corresponding proteins are shown on the right panel using different colors on behalf of specific motifs with the Multiple Em for Motif Elicitation (MEME)

Multiple sequence alignment of watermelon kinesin protein sequences was performed and analyzed using MEME online software to explore sequence features and functional motifs of each ClKIN protein. Seven typical conserved motifs for kinesin family proteins, named as motifs 1–7, have been identified (Fig. 4). Motif 1, highly conserved peptide sequence (FAYGQTGSGKT) inside the ATP-binding site and motif 6, a conserved microtubule-binding site (SSRSH), were found in all watermelon kinesins. Motif 4, another conserved microtubule-binding site ‘VDLAGSE’, could be detected in most ClKINs with the exception of ClKIN7C, ClKIN10D, ClKIN14A and ClKIN14C [32]. The motif 2, the microtubule-binding site ‘HIPYR’ existed in most ClKINs with the exclusion of ClKIN1D, ClKIN7C, ClKIN10D, ClKIN12A, ClKIN14M and ClKIN14G. Motif 3 is a conserved motif of K/RxIxNxxxVIN at the beginning of the neck region. Motif 5 is the highly conserved neck motif consisting of a hydrophobic repeat pattern of ø-xx(x)- ø-xxx-ø-xx-ø-G. Motif 3 and Motif 5 were presented in the majority of ClKINs [33]. The results showed that ClKINs proteins contained the typical conserved feature motifs of kinesin family.

### Duplication and syntenic analysis of kinesin gene families

**Fig. 5:**
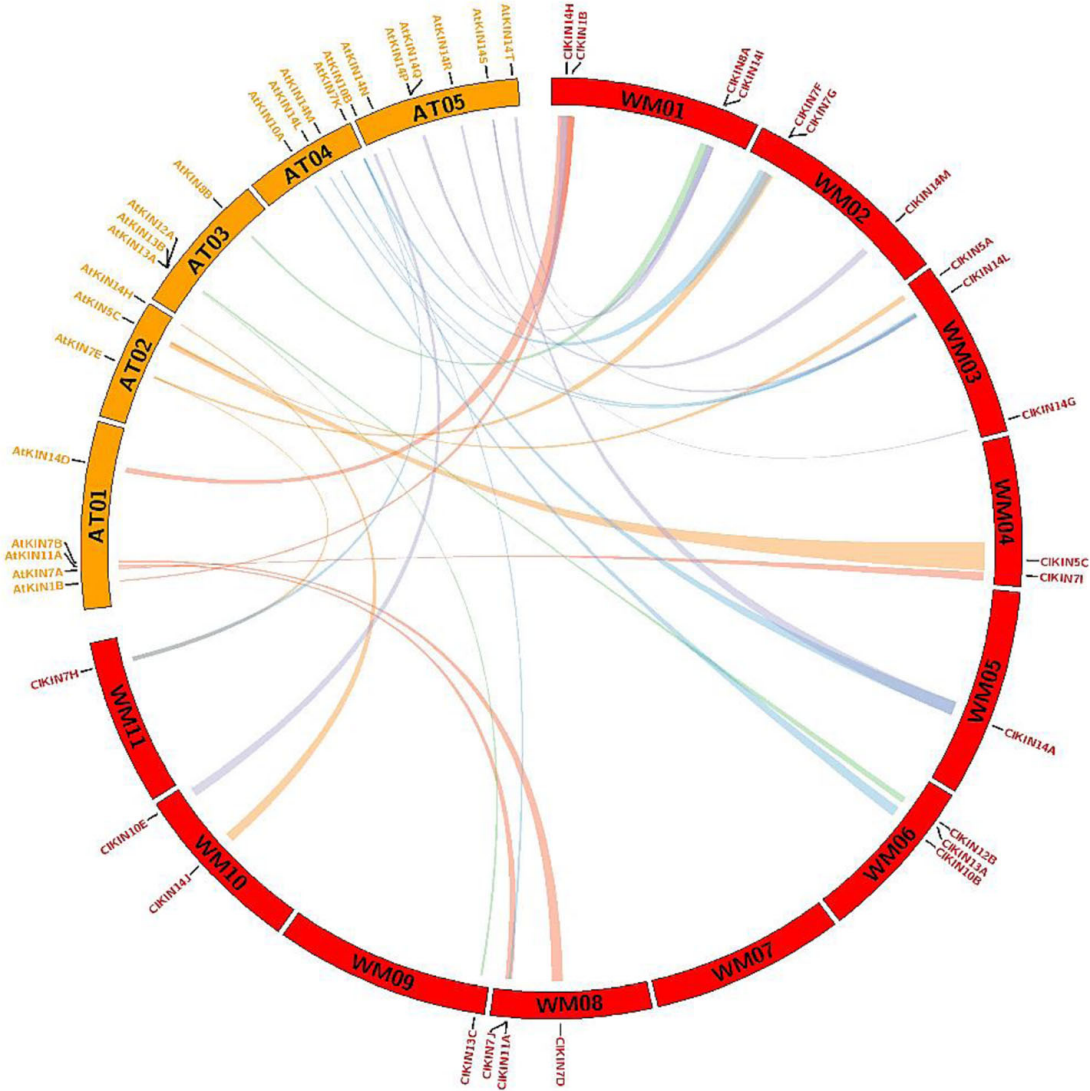
Synteny analysis between watermelon and Arabidopsis kinesin genes. Chromosomes of watermelon and Arabidopsis are shown in different colors (red and yellow) and in partial circles. The approximate distribution of each kinesin gene is presented by short black line on the circle. Colored curves indicate the syntenic relations between watermelon and Arabidopsis kinesin genes. The prefixes ‘WM’ and ‘AT’ respectively indicate watermelon *Citrullus lanatus* and *Arabidopsis thaliana*

Tandem and segmental duplications play important roles in the expansion and function of a gene family [34, 35]. To reveal the possible evolutionary relationships of kinesin gene families, duplication events, segmental and tandem duplication gene pairs of the kinesin family were investigated in *C. lanatus* and *A. thaliana*. The results implied that there are no tandem genome duplication events occurred for kinesin genes. However, 15 pairs of segmental duplication events were identified, where each pair of genes were situated at separate chromosome in watermelon genome, such as *ClKIN1B/ ClKIN1D*, *ClKIN7F/ClKIN7J*, *ClKIN14L/ ClKIN14A*, *ClKIN13B/ ClKIN13C* (Fig. 5). Overall, the synteny analyses suggested that the kinesin family in watermelon expanded only through segmental duplications.

### Expression profiles of kinesin genes in different tissues in watermelon

**Fig. 6:**
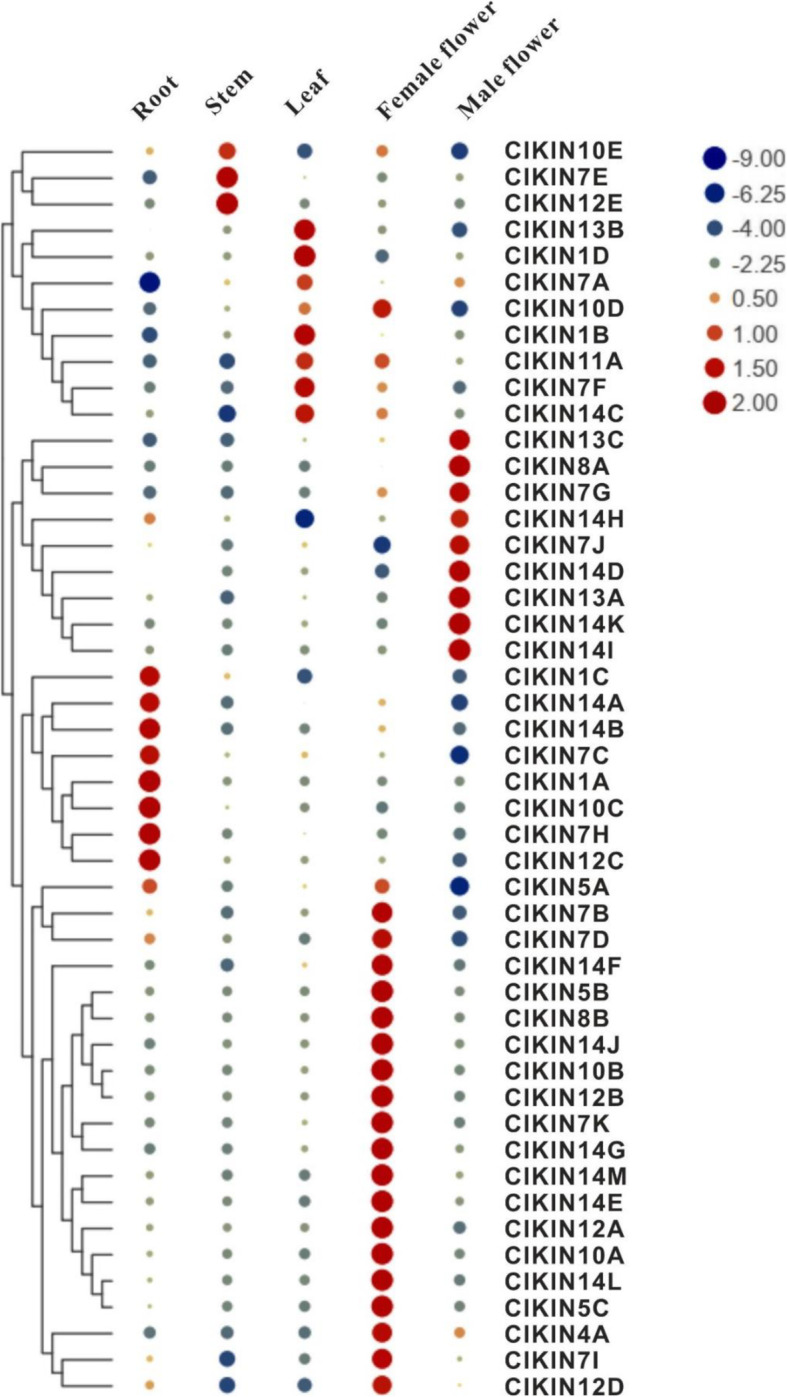
Expression profiles analysis of kinesin family in different tissues in watermelon. The data were showed as means value ±SD. All experiments were performed with three independent replicates. Log_2_-transformed data were used for the cluster analysis (*n* = 3). The inset shows the colour legend used in the cluster representation (Log_2_ ratios). The red dots indicate the higher expression level, whereas the blue dots indicate the lower expression level. *ClACTIN* gene was used for normalization of quantitative qRT-PCR results

The expression pattern is important for assessing the potential roles of *ClKINs* genes in the processes of plant growth and development. Therefore, we examined the expression patterns of 48 *ClKINs* genes in five different tissues including the root, stem, leaf, female and male flower by quantitative reverse transcription PCR (qRT-PCR) (Fig. 6). The qRT-PCR results indicated that the kinesin genes in watermelon exhibited a restricted expression pattern and could only be detected in one or two tissue tested. Eight watermelon kinesin genes (*Cla013748, Cla001716, Cla003337, Cla011614, Cla000622, Cla008965, Cla016598* and *Cla010272*) were highly expressed in the root, stem and leaf vegetative organs, which indicates their potential roles in vegetative organs development. In addition, 20 kinesin genes were preferentially expressed in the female flower. And 9 kinesin genes were specifically expressed in the male flowers. Overall, 38 kinesin genes were abundantly expressed in the reproductive organs, suggesting that they play critical roles in the growth and development of reproductive tissues.

### Key kinesin genes identification involved in early fruit development

**Fig. 7:**
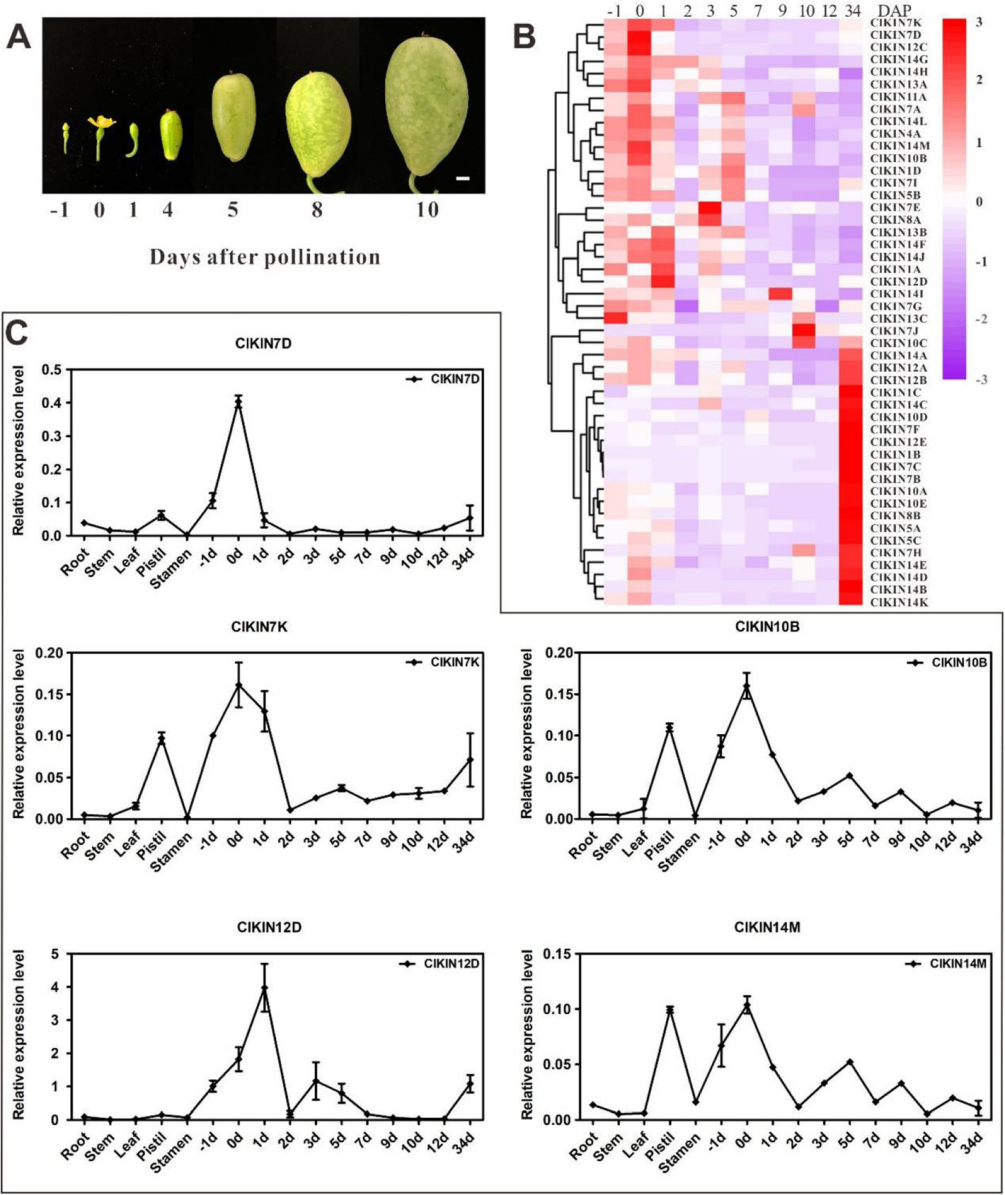
Expression profiles analysis of *ClKINs* genes during the early fruit development in watermelon. **a** The morphological characteristics of the watermelon fruits at different days after pollination. -1,0,1,4,5,8,10 respectively displays the days after pollination. **b** Expression patterns of *ClKINs* genes during the fruit development. Log_2_-transformed data were used for the cluster analysis (*n* = 3). The inset shows the colour legend used in the cluster representation (Log_2_ ratios). A red box indicates the higher expression level, whereas the purple box indicates the lower expression level. *ClACTIN* gene was used for normalization of quantitative qRT-PCR results. **c** The dynamic changes of expression levels analysis from the 5 *ClKINs* genes specifically or abundantly expressed in the early fruit development. The standard deviations of three biological replicates are represented by the error bars

**Fig. 8:**
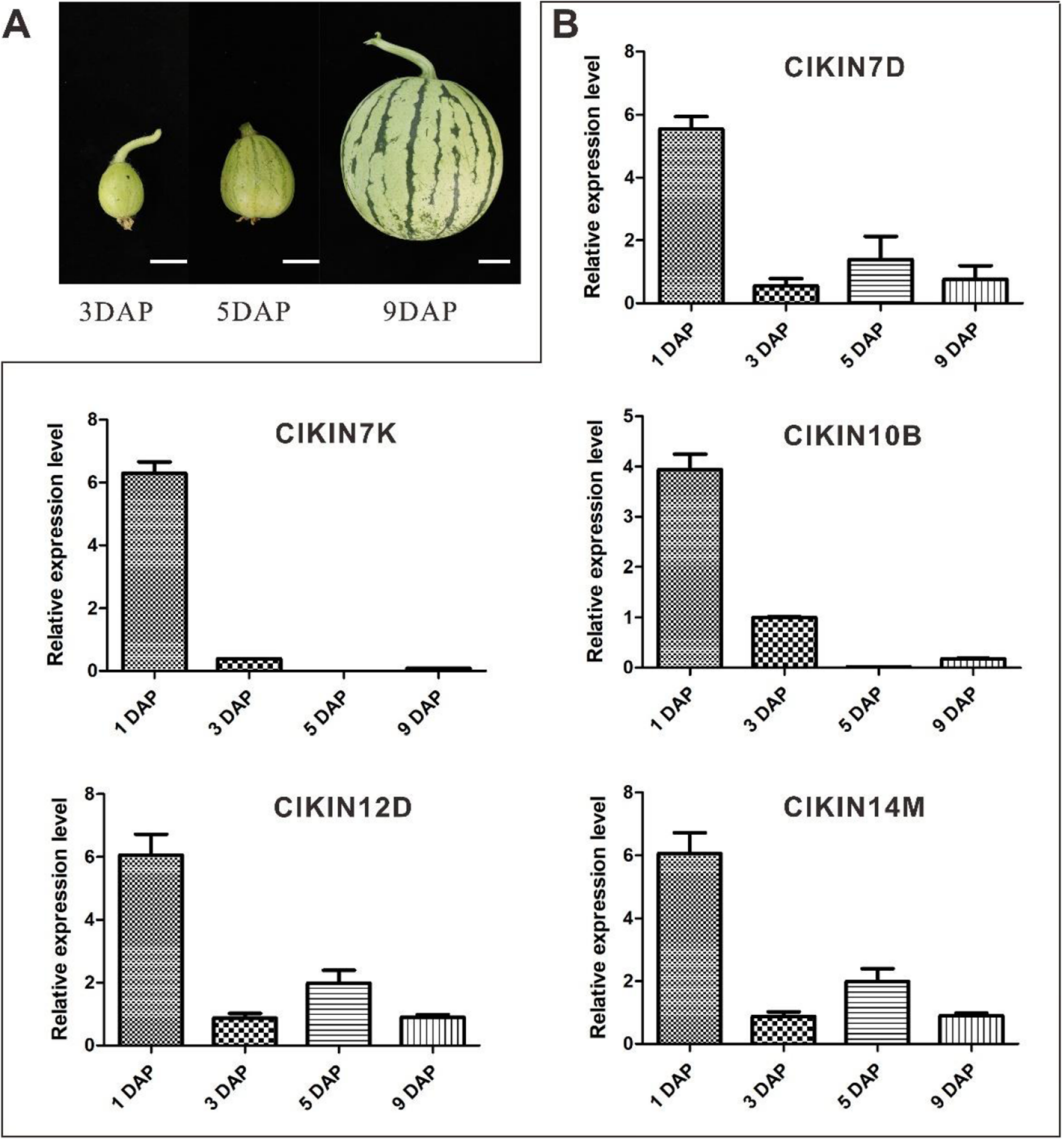
Expression profiles analysis of 5 *ClKINs* genes during the early fruit development in Chinese watermelon line 97,103 of round and medium-size fruit shape. **a** The morphological characteristics of the watermelon fruits at different days after pollination. 3, 5 and 9 DAP respectively display the days after pollination. **b** The expression levels analysis from the 5 *ClKINs* genes specifically or abundantly expressed in the early fruit development, especially in the fruit at 1DAP. The standard deviations of three biological replicates are represented by the error bars

Fruit development of watermelon, as a cucurbit species, follows the canonical developmental progression of four stages: ovary development; fruit set; expansive fruit growth; maturation and ripening [21, 22, 36]. Among the four stages, the first three stages of development (ovary development, fruit set, expansive fruit growth) are defined as the early fruit development stage. The early fruit development stage completes in about 10 days after pollination (DAP) and directly determines the size, shape and quality of fruit [37]. In addition, previous transcriptome and qRT-PCR analysis results demonstrated that kinesin family genes participated in the regulation of early fruit development in *Malus domestica* and *Cucumis sativus* [15, 17]. Therefore, to identify potential roles of kinesin family genes in the process of watermelon early fruit development, qRT-PCR was performed using cDNA prepared from the fruits at -1, 0, 1, 2, 3,5, 7, 9, 10, 12, 34 days after pollination (DAP). Hierarchical clustering and heatmap analysis were executed and gave a visual analysis of kinesin gene expression. From the overview of the kinesin expression profiles, the transcripts of all *ClKINs* genes tested could be detected in the fruit at different development stages, with different transcription levels at specific stage of fruit development (Fig. 7). Among them, 21 *ClKINs* showed high transcription levels in the fruits at 34 days DAP, which is at maturation and ripening development phase. A total of 27 *ClKINs* exhibited different expression levels in the fruits at-1, 0, 1, 2, 3,5, 7, 9, 10, 12 days DAP, which is at the early fruit development stage. Further detailed analysis revealed that 14 kinesins (*Cla022645*, *Cla013925*, *Cla010272*, *Cla009301*, *Cla019890*, *Cla007599*, *Cla014608*, *Cla014076*, *Cla000622*, *Cla018908*, *Cla014106*, *Cla015441*, *Cla022444* and *Cla008965*) showed specifically or abundantly expressed in the early developing fruits. Moreover, the major edible sections of watermelon fruit develop and differentiate from the pistil tissue. In consequence, comparative analysis of expression levels between the early fruit and the pistil tissue demonstrated that 5 kinesin *ClKINs* genes (*Cla022645*, *Cla013925*, *Cla019890*, *Cla007599* and *Cla018908*) showed relatively specific or highest expression level simultaneously both in the early developmental fruit and pistil. To better associate the functions of kinesin gene family in the early fruit development, we also examined the expression levels of 5 *ClKINs* genes in Chinese watermelon 97,103 which is round and medium-size fruit shape comparing to watermelon YL with the long and big fruit shape by qRT-PCR. The results indicated that 5 *ClKINs* genes abundantly expressed in the early fruit development stage, especially in the fruits at 1 DAP (Fig. 8). The expression of 5 *ClKINs* genes were further analyzed in seeds at different DAP. And 5 *ClKINs* genes showed different expression levels in seeds. Especially, *ClKIN7D* and *ClKIN12D* showed higher expression levels in seeds at 6 and 8 days after pollination (Figure S4). The results suggested that 5 *ClKINs* genes (*ClKIN7D*, *ClKIN7K*, *ClKIN10B*, *ClKIN12D* and *ClKIN14M*) are involved in fruits/seed development during the early fruit development.

**Fig. 9:**
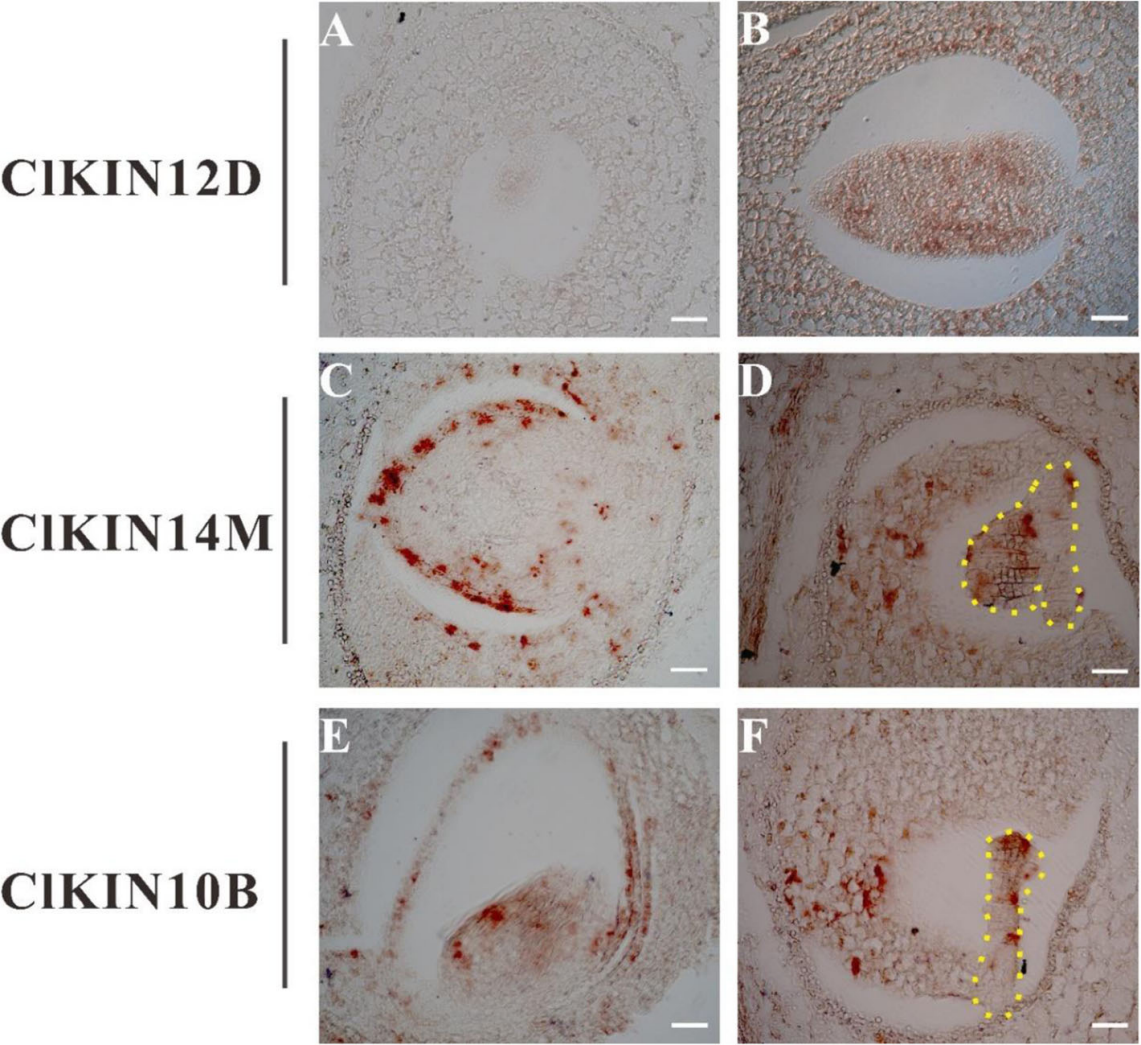
RNA in situ hybridization of *ClKIN10B*, *ClKIN12D* and *ClKIN14M* in early fruits/seeds. Positive signals (shown in red) are clearly restricted to the seed coat (**b**, **c** and **e**) and embryos (**d** and **f**) of the early fruits. When hybridized with sense probes, no signal is observed (**a**). The yellow breakpoint lines marked the embryos. Bars = 40 μm

In order to accurately examine *ClKINs* genes tissue expression patterns and verify genes functions, 3 of the above 5 genes (*ClKIN10B*, *ClKIN12D* and *ClKIN14M*) were selected for further verification by in situ hybridization assay. Detailed analysis showed that *ClKIN10B*, *ClKIN12D* and *ClKIN14M* have strong expression in early fruit/seeds development (Fig. 9). All these data indicated that the different kinesin members displayed diverse expression patterns and may have stage-specific roles during watermelon fruit/seed development. Especially, the 3 kinesin genes tested may function in the process of watermelon early fruit/seed development.

### Potential roles of kinesin family genes in response to hormone treatments

**Fig. 10:**
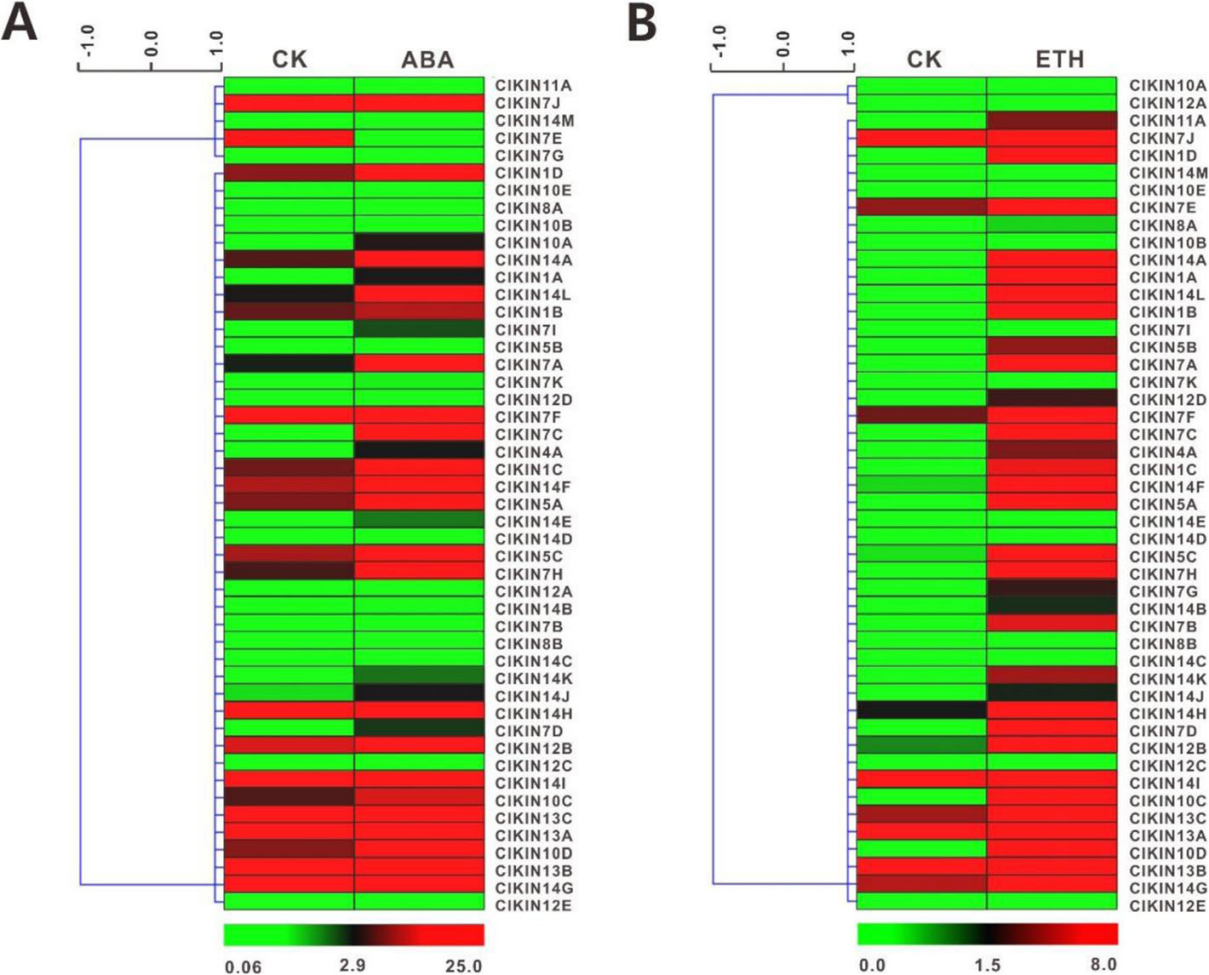
Expression levels analysis of kinesin family in watermelon under ABA and ETH hormones treatments. **a** Expression profiles of *ClKINs* genes under ABA stress treatment visualized as a heat map. **b** Expression levels of *ClKINs* genes under ETH stress treatment. The relative transcript levels were Log_2_ transformed and visualized as a heatmap via Mev6.0, using red to indicate increased expression level and green to indicate decreased expression level (displayed at the bottom)

To understand the possible relationship between watermelon kinesin genes and major hormones, the relative transcriptional levels of each kinesin gene were investigated after ABA and ETH hormones treatments. The heat map was created based on the relative expression levels (Fig. 10). The results revealed that at least 1/3 of kinesin genes were responsive to ABA or ETH treatments. Moreover, 23 *ClKINs* genes were regulated by both ABA and ETH, but showing very different expression pattern under different hormone treatment. Following ABA treatment, the expression levels of two *ClKINs* (*ClKIN11A* and *ClKIN7G*) were sharply down-regulated (< twofold), whereas the expression levels of 21 *ClKINs* significantly increased (> twofold). However, unlike ABA treatment, most of kinesin genes (32 *ClKINs*) exhibited significant up-regulation in response to ETH stimuli. After ETH treatment, the expression levels of *ClKIN7K*, *ClKIN10B* and *ClKIN14M* were not changed abruptly, however, *ClKIN7D* and *ClKIN12D* exhibited significant up-regulation (> twofold), and interestingly, *ClKIN7D* was also sharply up-regulated (< twofold) after ABA treatment. The results implied that *ClKIN7D* and *ClKIN12D* potentially involved in the regulation of the plant hormones pathway. In general, these detailed expression level analyses implied that *ClKINs* genes could participate in the regulation of the plant hormones pathway.

## Discussion

Watermelon [*Citrullus lanatus* (Thunb.) Matsum. & Nakai] comprises the major cucurbits and is the fifth consumed fruit in the world. It is also one of the most important economic crops grown worldwide. Fruit development traits are the very important agronomic traits in watermelon breeding. The early fruit development in watermelon directly affects the subsequent agronomic traits, including the fruit size, shape and quality. Kinesins are important microtubule-based motor proteins with conserved motor domains among all eukaryotic organisms. They play critical roles in the unidirectional transport of vesicles and organelles, cytokinesis, signal transduction, morphogenesis, cell division and cell growth in the plant development [38–41]. Furthermore, previous researches have revealed that the kinesin family genes also participated in plant reproductive development [29–31, 42]. Especially, kinesin family genes have been proved to be essential for the regulation of early fruit development in *Malus domestica* and *Cucumis sativus* [15, 17]. Therefore, these works declared the urgency of extensively investigation of kinesin family genes in plants, especially in economic crops, with the expectation to improve crop yield. However, the identification and analysis of detailed expression characteristics and functions of kinesin family genes in watermelon, especially in the fruit reproductive tissues, still remain elusive. With the completion of the *C. lanatus* genome sequence, the *ClKINs* genes can be systematically identified and analyzed [43]. In the present study, we have identified 48 kinesin family genes *ClKINs* in the watermelon genome and comprehensively analyzed these genes for their phylogenetic relationships, chromosomal locations, gene structures, conserved motif distributions, and duplication and syntenic analysis. In addition, we performed the extensive analysis of *ClKINs* expression patterns in different tissues, at different early fruit developmental stages and in response to hormones treatments. Especially, expression patterns analysis during fruit developmental process elaborated the overall characteristics and the specific dynamics of watermelon kinesin family genes in watermelon development. Conclusively, our work provides clear clues to further investigation of their detailed roles in watermelon reproductive development and response to hormones influence.

### Characteristics of kinesin family genes in watermelon

As described above in results section, some typical conserved motifs for kinesin family proteins exists in most watermelon kinesins. Kinesin motor domain is comprised of a Walk A ATP binding motif “FAYGQTGSGKT” and a microtubule binding domain [44–46]. Microtubule binding domain commonly contains three microtubule binding motifs (SSRSH, xDLAGSE and HxPYR) [32]. Highly conserved peptide sequence “FAYGQTGSGKT” in the ATP-binding motif could be found in all of watermelon kinesins through the alignments of their amino acid sequences, which is responsible for hydrolyze ATP to produce a direct force to travel unidirectionally along microtubule protofilaments and power multiple critical cellular process. The typical microtubule binding site “SSRSH” could be detected in all of kinesins, but the other two microtubule binding motifs “xDLAGSE and HxPYR” could be found in most of kinesins with few exceptions. In particular, only one microtubule binding motif “SSRSH” could be detected in two kinesins ClKIN7C and ClKIN10D, but the other two of three microtubule binding motifs couldn't be found. The results suggested that the microtubule binding site “SSRSH” is the most conserved microtubule binding site. However, whether ClKIN7C and ClKIN10D proteins possess the microtubule binding abilities with only one microtubule binding site needs further verification.

Phylogenetic analysis based on kinesin protein sequences categorized the kinesin genes from watermelon, *Arabidopsis* and rice into ten families [47]. Interestingly, the number distribution tendency of all these genes from three species was almost the same in the ten groups and did not show distinct monocot or dicot distribution characteristics. The analysis of the consistent tendency showed that the kinesin-14 and kinesin-7 respectively were the first and the second largest group. The kinesin-14 family was one well-conserved family and played important roles in chromosome segregation at mitosis and organelle transport [48]. The number of kinesin-14 family members is the maximum both in animals and plants [48, 49]. The kinesin-11 subfamily contained the minimum amount of kinesin protein, which had only one kinesin-11 protein in each of the three species. Kinesin-11 family members function in signal transduction or divergent catalytic core and are rarely found. In addition, because the kinesin-2, 3, and 9 subfamilies are absent from land plants, the watermelon kinesin superfamily lacks the three subfamilies [50]. The results of phylogenetic analysis implied that kinesin family genes were spatially and functionally conserved in some essential developmental processes in different plant taxa.

### Essential roles of kinesin family genes in early fruit development

Previous microarray and expression profiling analysis have revealed that some kinesin genes were necessary for early fruit development in apple and cucumber [15, 17, 18, 51]. However, the exact roles of kinesin genes in the process of early fruit development are still unknown in most economic species, including watermelon. Therefore, the expression profiles of kinesin family genes in early fruit development were comprehensively analyzed. qRT-PCR results demonstrated that the transcripts of most kinesin genes could be detected in early developmental fruits at different development stages. A striking feature was that the expression levels of most kinesin genes were higher in fruits at early development stages and then sharply decreased at fruit maturation and ripening. Studies of early fruit development in cucurbits showed that the early fruit growth is primarily due to cell number increments, or in the other words, primarily driven by cell division [52]. Moreover, the period of rapid cell division was accompanied by increased peak expression of microtubule related kinesin genes [18]. Microtubules facilitate alignment of chromosomes at the spindle equator in mitosis [53]. Therefore, the high expression level of kinesin genes in the early fruit development stages may regulate chromosome organization during mitosis via regulation of cytoskeleton and microtubule dynamics and finally caused the change of the cell amounts or sizes [53].

Which developmental process do the watermelon kinesin genes regulate to ultimately control the early fruit development? This question is intriguing and needs to be characterized in future. The watermelon fruit as well as tomato fruit are classified as a berry fruit because the thick pericarp encloses many seeds. The edible parts of two kinds of fruits are either mainly composed of placentas or develop and differentiate from the placenta tissues [21]. The comprehensive tissue-specific transcriptome analysis revealed that the kinesin gene *SpPAKRP1* showed peak expression in the placenta tissue during the early stage of fruit development in *Solanum pimpinellifolium*, a wild cultivated tomato [19, 20]. This suggested that the tomato kinesin gene *SpPAKRP1* could be involved in the early fruit development by regulating the placenta tissue development. This implies that the watermelon kinesin genes *ClKINs*, specifically or abundantly expressed in the early fruit development stage, could also control the early fruit development via regulating the placenta tissue development. This provided an ideal entry point to study the molecular mechanism of early fruit development through analyzing the role in the placenta development. Nevertheless, the exact roles of these kinesin genes need to be further studied and confirmed.

### Potential roles of kinesin family genes in response to hormones treatments

Plant hormones are a group of small signal molecules which have been approved to play essential roles in different processes of plant growth and development. The expression levels of a larger number of genes are known to regulated by different plant hormones. Previous studies have discovered the contribution of hormones such as ethylene (ETH) in sex determination and development of sex-specific floral organs in the Cucurbitaceae [54, 55]. In addition, study has indicated that kinesin-4 gene *OsGDD11* is involved in the signaling pathways of plant hormone [27]. More importantly, in cucurbits crops, pollination was believed to be the key process to release hormonal enzymes, most specifically auxin, which in turns to stimulate fruit enlargement [23]. Early studies demonstrated that plant hormones have been implicated to facilitate early fruit development in cucurbits, although there is debate as to which hormones are most critical [23, 56]. Mitotic kinesins play important roles in chromosome organization during mitosis in developing cucurbits cucumber fruits [17, 53]. These studies implied that there is a connection between plant hormones and kinesins. The fact has been first verified in rice, in which kinesin protein gene *BC12/GDD1* mediated cell elongation by regulating the hormone GA biosynthesis pathway [27]. In order to further explore the relationships between plant hormones and kinesins in watermelon, in our present work, the relative expression levels of kinesin genes after hormones treatments were investigated. The results showed that the transcription levels of most kinesin genes changed after hormones treatments, indicating their critical roles in response to different hormones. Although some genes could respond to the same hormones, some other members of kinesin family genes showed their roles differentially in response to certain hormones. Interestingly, *ClKIN7E* and *ClKIN7G* were down-regulated after ABA treatment, which is quite different from other kinesin genes, implying their unique roles in response to ABA hormone treatment. Taken together, the data provided useful clues for the further investigations of molecular mechanism of kinesins in response to plant hormones during the plant development process.

## Conclusions

In conclusion, 48 *ClKINs* genes were identified in *C. lanatus* at the whole-genome level. These genes were categorized into 10 subfamilies. The chromosomal locations, exon/intron structures, conserved motif distributions, and syntenic analysis of kinesin family members in *C. lanatus* were determined. Comprehensive analysis and expression profiling of *ClKINs* genes were performed to determine the potential functions in early fruit development and in response to hormones stimuli. Furthermore, detailed expression analysis revealed the tissue-specific and highly expression pattern of *ClKINs* genes. Finally, 5 *ClKINs* genes, including *ClKIN7D*, *ClKIN7K*, *ClKIN10B*, *ClKIN12D* and *ClKIN14M*, demonstrated relatively specifically and highest expression level simultaneously in the early fruit developmental, indicating their important roles in the early fruit developmental.

## Methods

### Identification of kinesin gene family in *Citrullus lanatus*

All BLAST searches were conducted in the watermelon genome database (Cucurbit Genomics Database, http://www.icugi.org/) by using three motor domain sequences from the KHC (N-terminus motor in human), KIF2 (internal motor in mouse), and KCBP (C-terminus motor in *Arabidopsis*) as queries. Sixty-three candidate genes generated by using an E-value cut-off of 1, which contained kinesins and some unrelated proteins. In addition, Hidden Markov Model (HMM) profiles of the motor domain (PF00225) was downloaded from the Pfam database (http://pfam.xfam.org/). Then HMMER 3.0 software was used to search for kinesins. Motor domain analysis in SMART (http://smart.embl-heidelberg.de/) and INTERPROSCAN (http://www.ebi.ac.uk/interpro/) were performed and the proteins without conserved motor domain were deleted. Finally, the candidate kinesin genes in watermelon were further analyzed with the online tools ExPASY (http://www.expasy.org/tools/) to predict the isoelectric point (PI) and molecular weight (MW).

### Chromosome localization analysis of watermelon kinesin genes

The chromosome locations information of all *ClKINs* genes were downloaded from watermelon genomics database. The information, including localizations and length of the chromosomes, were visualized by MapChart online software.

### Phylogenetic analysis of kinesin genes

Kinesin amino acid sequences from *C. lanatus* with *A. thaliana*, *O. sativa* and *Solanum lycopersicum* were aligned using the software Muscle with the default multiple alignment parameters. The phylogenetic trees were constructed via MEGA 6.06 using the neighbor joining method. The bootstrap replicates test value was set as 1000.

### Gene structures and conserved motifs analysis of kinesin proteins

The *ClKINs* gene structures were visualized by using the program GSDS2.0 (Gene Structure Display Server, http://gsds.cbi.pku.edu.cn/). The conserved motifs in *C. lanatus* kinesin proteins were identified by using the program MEME (Multiple Em for Motif Elicitation, http://meme-suite.org/tools/meme). The maximum number of motifs was set to 7 and the others were default.

### Syntenic analysis of watermelon *ClKINs* genes

The homolog pairs between *C. lanatus* and *A. thaliana* were identified using the BLASTp program. GFF files serves as input documents for MCScanX to analyze the synteny relationship [57]. The analysis result was visualized using the software CIRCOS (http://circos.ca/).

### Plant materials and hormones treatments

97,103 is an inbred line provided by Dr. Yong Xu from Beijing Key Laboratory of Vegetable Germplasm Improvement. YL watermelon materials were collected from the desert area in Yulin, Shaanxi Province, China. Under normal conditions, various tissues including root, stem, leaf, female flower, male flower and fruits at different days after pollination of *C. lanatus* were collected for RNA extraction. The watermelon plants were grown under natural light with temperatures of 28–35 °C/16–20 °C (day/night) in a greenhouse in spring. For hormones treatments, four-week-old seedlings after sowing were used phytohormones treatments. The leaves of the seedlings were sprayed with 100 μM Abscisic acid (ABA) [58] and 10 mM Ethephon (ETH) [59] and collected after 12 h treatments. The control seedlings were sprayed with the same solutions except for corresponding hormones. The taken samples with three biological replicates were immediately frozen in liquid nitrogen and stored at -80 °C before RNA extraction.

### RNA extraction and qRT-PCR

The total RNA of virous tissues were extracted using the Quick RNA isolation kit (Huayueyang Biotechnologies Co. Ltd, Beijing, China) according to the manufacturer’s instructions. The first-strand cDNA was synthesized with 1 μg total RNA using SuperScript III transcriptase (Invitrogen).

Quantitative reverse transcription PCR (qRT-PCR) was conducted on an ABI StepOnePlus machine using SYBR Premix Ex Taq™ (TaKaRa). Three independent biological repeats were performed for each *ClKIN* gene. Specific primers for all *ClKINs* genes were designed using Primer3Plus online software (http://www.bioinformatics.nl/cgi-bin/primer3plus/primer3plus.cgi) and listed in Supplementary Table S2. The relative expression levels of *ClKINs* genes were normalized against that of the watermelon *ACTIN* gene (gene ID: *Cla007792*) transcript.

### RNA in situ hybridization

To analyze the tissue expression patterns of kinesin genes in fruits at the early fruit development stage, 2-, 3- and 5-DAP fruits of watermelon YL line were used for RNA in situ hybridization as described in our previous work [42]. The fruits were fixed for 16 h in 4% paraformaldehyde solution with 0.1% Triton X-100 and 0.1% Tween 20 in PBS. After dehydration using graded ethanol and vitrification by dimethylbenzene, the samples were embedded in paraffin. The paraffin blocks were cut into 8 μm thick sections. The reaction results of in situ hybridization signals were detected as purple red color by adding the substrates nitroblue tetrazolium/5-bromo-4-chloro-3-indolyl-phosphate (NBT/BCIP). The hybridization signals were observed and photographed with an Axio imager M2 microscope (Zeiss).

## Supplementary Information

**Additional file 1**: **Figure S1**. Phylogenetic tree of kinesin genes from *C. lanatus*using neighbor-joining method. **Figure S2**. Phylogenetic tree of kinesin genes from *C. lanatus*and and *A. thaliana *using neighbor-joining method. **Figure S3**. Phylogenetic tree of kinesin genes from *C. lanatus*and and *S. lycopersicum *using neighbor-joining method. Neighbor-joining phylogenetic tree of the kinesin family. The different kinesin subfamily was marked with different color, respectively. **Figure S4**. Comparative expression analysis of 5 *ClKINs*genes in seed at different days after pollination by qRT-PCR. *ClACTIN* gene was used for normalization of quantitative RT-PCR results. The standard deviations of three biological replicates are represented by the error bars. **Table S1**. Kinesin gene family members identified in *Citrullus lanatus. ***Table S2**. The specific primers for qRT-PCR of 48 *ClKINs*genes.

## Data Availability

The datasets generated during the current study are available in the GenBank repository, http://www.ncbi.nlm.nih.gov/Genbank and the accession numbers are as follows: ClKIN1A (MW882942), ClKIN1B (MW882943), ClKIN1C (MW889992), ClKIN1D (MW889993), ClKIN4A(MW889994), ClKIN5A(MW889995), ClKIN5B (MW889996), ClKIN5C (MW889997), ClKIN7A(MW889998), ClKIN7B(MW889999), ClKIN7C(MW890000), ClKIN7D(MW890001), ClKIN7E(MW890002), ClKIN7F (MW958182), ClKIN7G (MW958183), ClKIN7H (MW958184), ClKIN7I (MW958185), ClKIN7J (MW958186), ClKIN7K (MW958187), ClKIN8A (MW958188), ClKIN8B (MW958189), ClKIN10A (MW958190), ClKIN10B (MW958191), ClKIN10C (MW958192), ClKIN10D (MW958193), ClKIN10E (MW958194), ClKIN11A (MW958195), ClKIN12A (MW958196), ClKIN12B (MW958197), ClKIN12C (MW958198), ClKIN12D (MW958199), ClKIN12E (MW958200), ClKIN13A (MW958201), ClKIN13B (MW958202), ClKIN13C (MW958203), ClKIN14A (MW958204), ClKIN14B (MW958205), ClKIN14C (MW958206), ClKIN14D (MW958207), ClKIN14E (MW958208), ClKIN14F (MW958209), ClKIN14G (MW958210), ClKIN14H (MW958211), ClKIN14I (MW958212), ClKIN14J (MW958213), ClKIN14K (MW958214), ClKIN14L (MW958215), ClKIN14M (MW958216).

## Abbreviations

*Cl*: *Citrullus lanatus*
KIN: Kinesin
MT: Microtubule
qRT-PCR: Quantitative reverse transcription PCR
PAKRP: Phragmoplast-associated kinesin-related protein
