# Supplementary material for: Genome wide analysis of kinesin gene family in Citrullus lanatus reveals an essential role in early fruit development
Source: BMC Plant Biol. 2021 May 10;21:210. doi: 10.1186/s12870-021-02988-6 (PMC8108342; doi:10.1186/s12870-021-02988-6)
Supplement: Supplementary file 1 — Additional file 1: Figure S1. Phylogenetic tree of kinesin genes from C. lanatususing neighbor-joining method. Figure S2. Phylogenetic tree of kinesin genes from C. lanatusand and A. thaliana using neighbor-joining method. Figure S3. Phylogenetic tree of kinesin genes from C. lanatusand and S. lycopersicum using neighbor-joining method. Neighbor-joining phylogenetic tree of the kinesin family. The different kinesin subfamily was marked with different color, respectively. Figure S4. Comparative expression analysis of 5 ClKINsgenes in seed at different days after pollination by qRT-PCR. ClACTIN gene was used for normalization of quantitative RT-PCR results. The standard deviations of three biological replicates are represented by the error bars. Table S1. Kinesin gene family members identified in Citrullus lanatus. Table S2. The specific primers for qRT-PCR of 48 ClKINsgenes. [file 12870_2021_2988_MOESM1_ESM.doc]

**Genome wide analysis of kinesin gene family in *Citrullus lanatus* reveals an essential role in early fruit development**

Shujuan Tian^#^, Jiao Jiang^#^, Guo-qi Xu, Tan Wang, Qiyan Liu, Xiner Chen, Man Liu, Li Yuan*****

State Key Laboratory of Crop Stress Biology for Arid Areas, College of Horticulture, Northwest A&F University, Yangling, 712100, Shaanxi, China

**Supplementary materials**


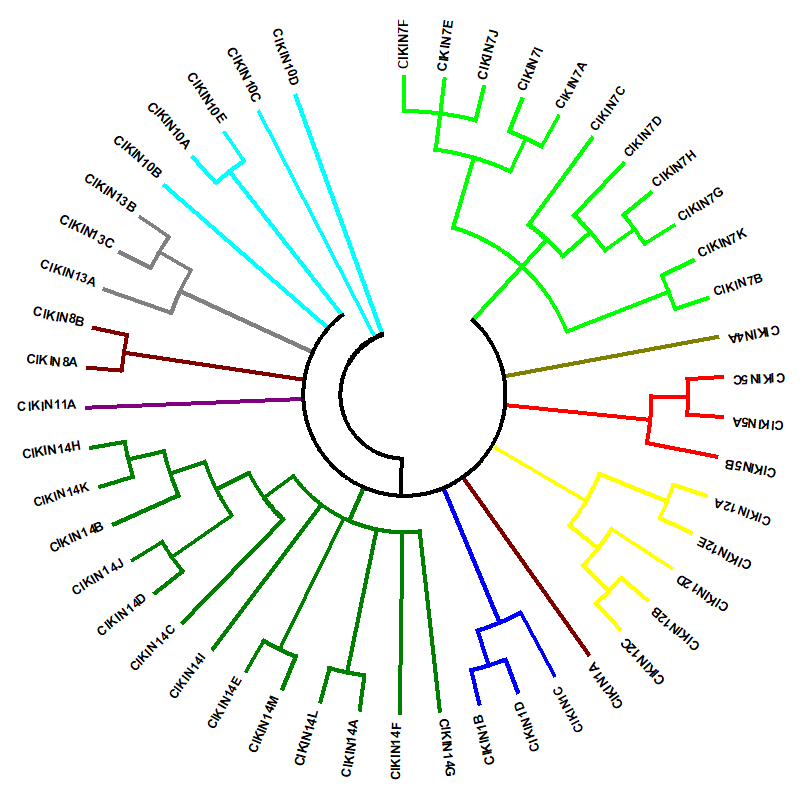


**Figure S1.** Phylogenetic tree of kinesin genes from *C. lanatus* using neighbor-joining method.


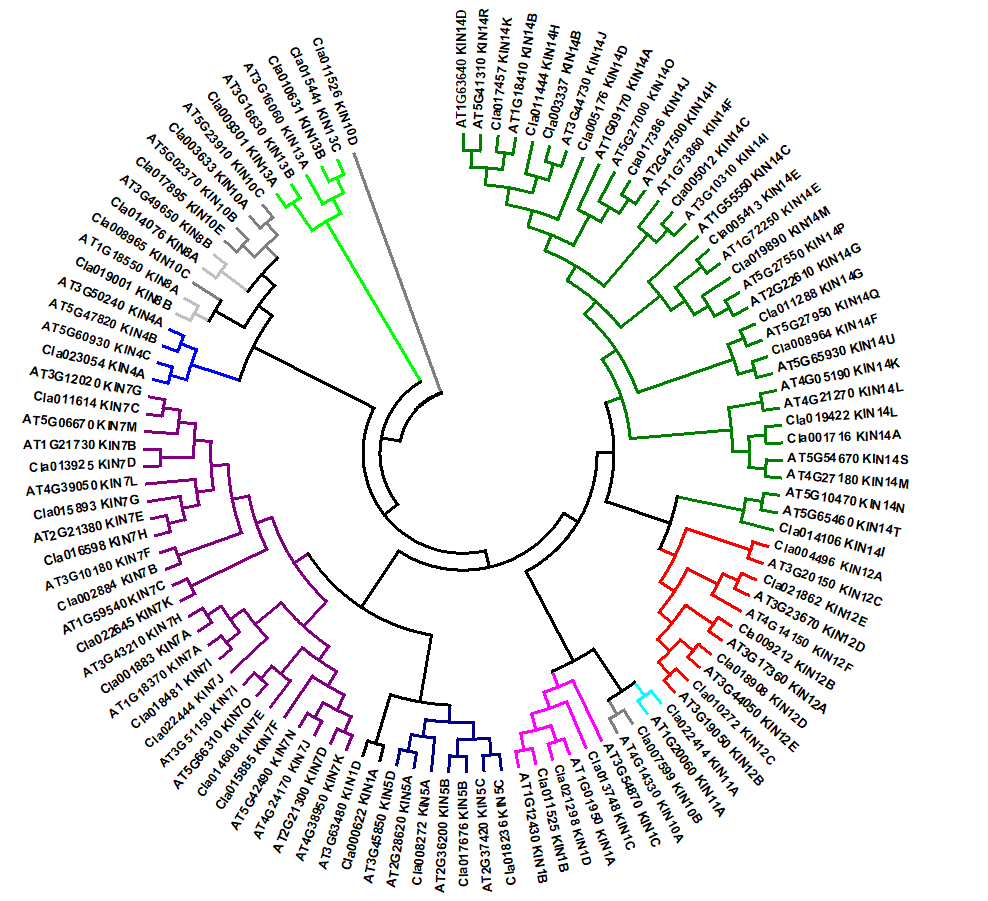


**Figure S2.** Phylogenetic tree of kinesin genes from *C. lanatus* and and *A. thaliana* using neighbor-joining method.


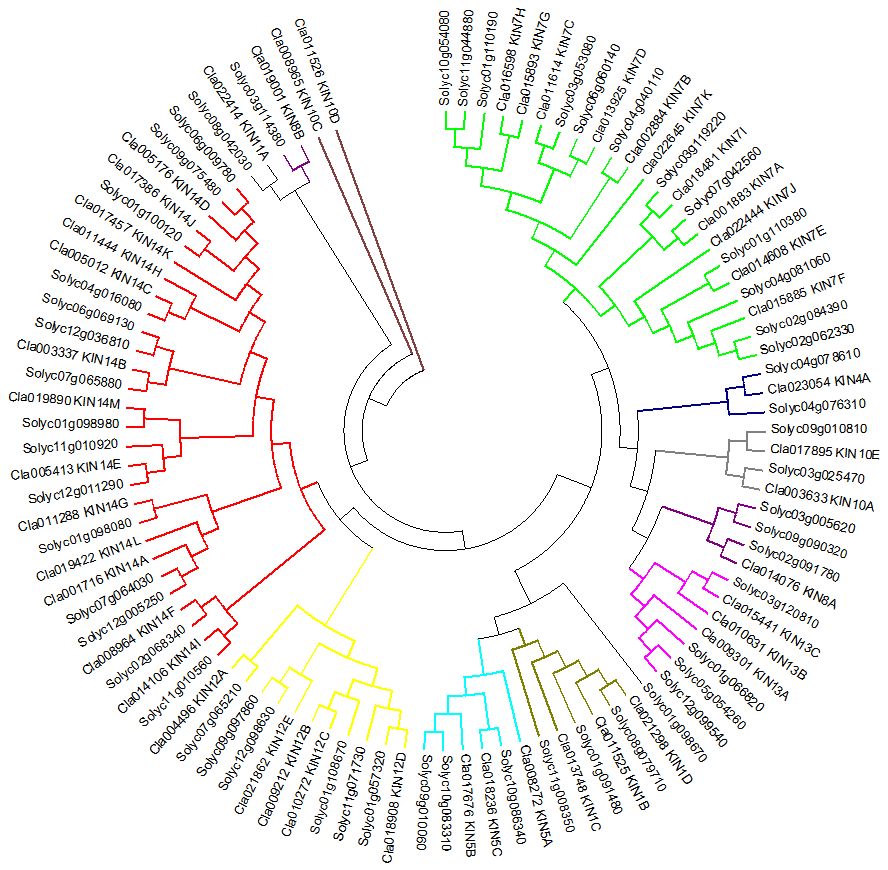


**Figure S3. Phylogenetic tree of kinesin genes from *C. lanatus* and and *S. lycopersicum* using neighbor-joining method.** Neighbor-joining phylogenetic tree of the kinesin family. The different kinesin subfamily was marked with different color, respectively.


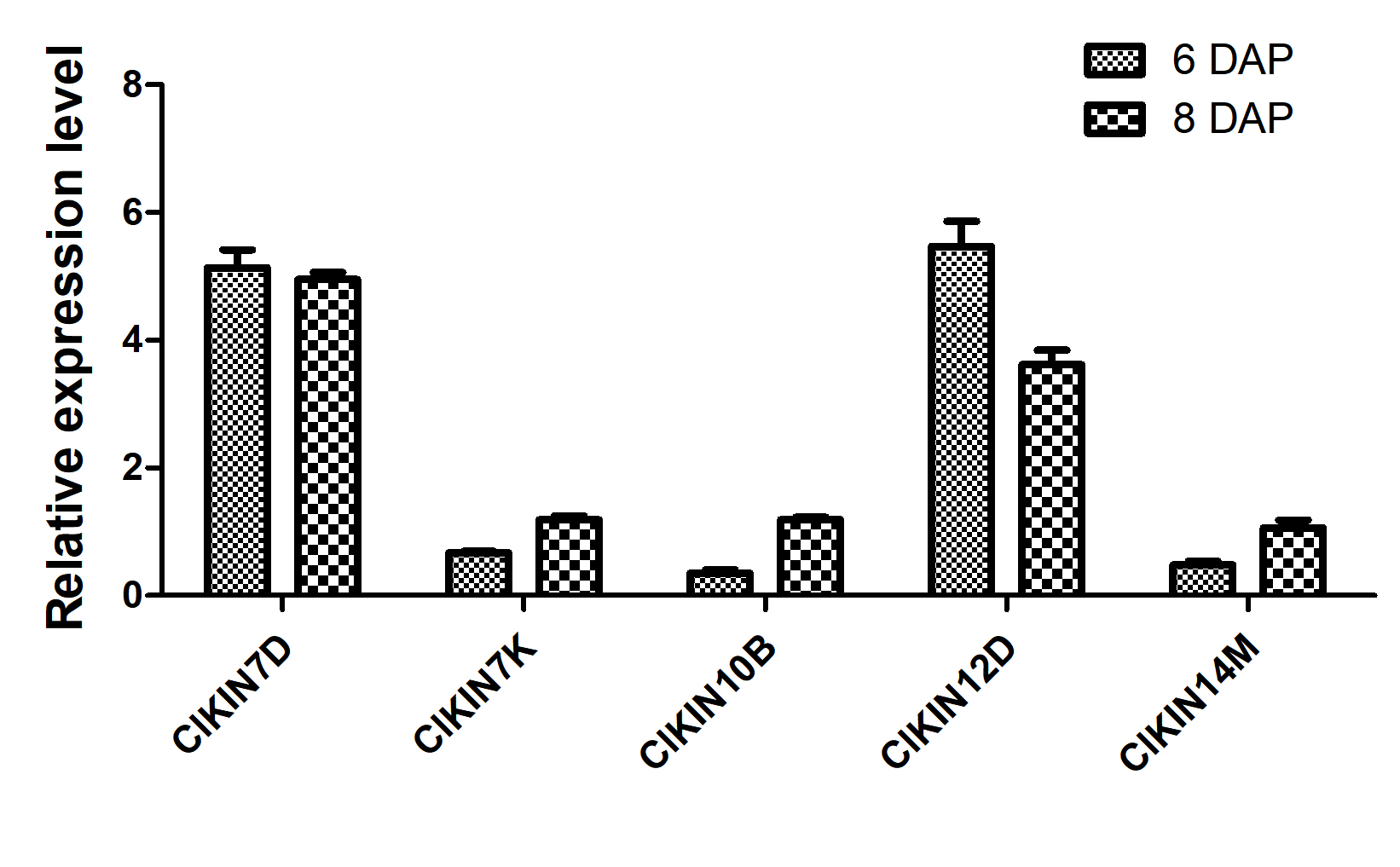


**Figure S4. Comparative expression analysis of 5 *ClKINs* genes in seed at different days after pollination by qRT-PCR.** *ClACTIN* gene was used for normalization of quantitative RT-PCR results. The standard deviations of three biological replicates are represented by the error bars.

**Table S1. Kinesin gene family members identified in *Citrullus lanatus***

| **Gene name** | **Gene ID** | **Length (Kb)** | **Amino Acids (aa)** | **No. of introns** | **No. of exons** | **Isoelectric point (pI)** | **Molecular Weight (KDa)** |
| --- | --- | --- | --- | --- | --- | --- | --- |
| *ClKIN1A* | *Cla000622* | 5.01 | 442 | 13 | 14 | 5.1 | 49.1 |
| *ClKIN1B* | *Cla011525* | 3.86 | 377 | 6 | 7 | 7.07 | 40.8 |
| *ClKIN1C* | *Cla013748* | 12.11 | 1002 | 19 | 20 | 7.18 | 112.4 |
| *ClKIN1D* | *Cla021298* | 10.28 | 870 | 17 | 18 | 6.31 | 96.6 |
| *ClKIN4A* | *Cla023054* | 10.7 | 1221 | 24 | 25 | 7.49 | 137 |
| *ClKIN5A* | *Cla008272* | 6.43 | 1051 | 21 | 22 | 5.65 | 118 |
| *ClKIN5B* | *Cla017676* | 6.86 | 1001 | 22 | 23 | 5.53 | 113 |
| *ClKIN5C* | *Cla018236* | 7.46 | 1051 | 22 | 23 | 5.8 | 118.4 |
| *ClKIN7A* | *Cla001883* | 11.89 | 941 | 12 | 13 | 8.55 | 106.4 |
| *ClKIN7B* | *Cla002884* | 21.5 | 1205 | 29 | 30 | 5.38 | 137.7 |
| *ClKIN7C* | *Cla011614* | 18.23 | 367 | 8 | 9 | 9.47 | 41.2 |
| *ClKIN7D* | *Cla013925* | 13.09 | 1146 | 23 | 24 | 6.72 | 128 |
| *ClKIN7E* | *Cla014608* | 10.29 | 696 | 11 | 12 | 5.38 | 78 |
| *ClKIN7F* | *Cla015885* | 7.66 | 962 | 12 | 13 | * | * |
| *ClKIN7G* | *Cla015893* | 10.6 | 1078 | 23 | 24 | 5.45 | 119.7 |
| *ClKIN7H* | *Cla016598* | 10.71 | 1083 | 23 | 24 | 6.14 | 120.1 |
| *ClKIN7I* | *Cla018481* | 5.12 | 960 | 13 | 14 | 6.92 | 109.3 |
| *ClKIN7J* | *Cla022444* | 7.38 | 946 | 11 | 12 | 6.04 | 107.1 |
| *ClKIN7K* | *Cla022645* | 6.29 | 830 | 16 | 17 | 5.83 | 94.2 |
| *ClKIN8A* | *Cla014076* | 7.74 | 716 | 14 | 15 | 7.2 | 80.3 |
| *ClKIN8B* | *Cla019001* | 4.75 | 757 | 5 | 6 | 9.65 | 84.4 |
| *ClKIN10A* | *Cla003633* | 6.83 | 700 | 15 | 16 | 7.95 | 76.5 |
| *ClKIN10B* | *Cla007599* | 4.66 | 837 | 5 | 6 | 5.8 | 93.1 |
| *ClKIN10C* | *Cla008965* | 12.67 | 892 | 16 | 17 | 6.01 | 102.1 |
| *ClKIN10D* | *Cla011526* | 5.18 | 419 | 9 | 10 | 6.41 | 46.4 |
| *ClKIN10E* | *Cla017895* | 4.61 | 616 | 11 | 12 | 8.61 | 67.6 |
| *ClKIN11A* | *Cla022414* | 10.13 | 835 | 18 | 19 | 5.94 | 93.6 |
| *ClKIN12A* | *Cla004496* | 6.94 | 1142 | 14 | 15 | 5.59 | 128.8 |
| *ClKIN12B* | *Cla009212* | 15.33 | 2202 | 29 | 30 | 5.21 | 250.1 |
| *ClKIN12C* | *Cla010272* | 16.07 | 2889 | 34 | 35 | 5.13 | 330.3 |
| *ClKIN12D* | *Cla018908* | 9.93 | 1439 | 21 | 22 | 5.12 | 162.7 |
| *ClKIN12E* | *Cla021862* | 9.26 | 1305 | 15 | 16 | 5.28 | 145.4 |
| *ClKIN13A* | *Cla009301* | 7.05 | 811 | 11 | 12 | 6.04 | 89.6 |
| *ClKIN13B* | *Cla010631* | 5.63 | 724 | 11 | 12 | 6.35 | 81.8 |
| *ClKIN13C* | *Cla015441* | 6.11 | 679 | 11 | 12 | 6.05 | 76.1 |
| *ClKIN14A* | *Cla001716* | 5 | 752 | 16 | 17 | 5.59 | 84.9 |
| *ClKIN14B* | *Cla003337* | 9.72 | 1148 | 16 | 17 | 8.67 | 127.4 |
| *ClKIN14C* | *Cla005012* | 17.45 | 858 | 14 | 15 | 9.11 | 95.5 |
| *ClKIN14D* | *Cla005176* | 7.51 | 1064 | 18 | 19 | 5.49 | 117.7 |
| *ClKIN14E* | *Cla005413* | 6.26 | 1117 | 16 | 17 | 6.42 | 124.9 |
| *ClKIN14F* | *Cla008964* | 3.68 | 308 | 4 | 5 | 8.77 | 35 |
| *ClKIN14G* | *Cla011288* | 4.16 | 632 | 9 | 10 | 9.33 | 71.3 |
| *ClKIN14H* | *Cla011444* | 10.84 | 1068 | 19 | 20 | 6.29 | 119 |
| *ClKIN14I* | *Cla014106* | 19.12 | 1276 | 22 | 23 | 5.99 | 141.2 |
| *ClKIN14J* | *Cla017386* | 6.97 | 1017 | 18 | 19 | 8.43 | 112.1 |
| *ClKIN14K* | *Cla017457* | 7.52 | 1336 | 19 | 20 | 5.85 | 150.4 |
| *ClKIN14L* | *Cla019422* | 5.99 | 773 | 15 | 16 | 7.51 | 87.1 |
| *ClKIN14M* | *Cla019890* | 5.03 | 810 | 10 | 11 | 9.49 | 89.8 |

***** show that there are continuous “Ns” in the ClKINs protein sequences, making impossible to calculate their PI and MW.

**Table S2. The specific primers for qRT-PCR of 48 *ClKINs*** genes

| **Gene name** | **Gene locus** | **Primer sequences (5’-3’)** | | **Product length(bp)** |
| --- | --- | --- | --- | --- |
|  |  |  |  |  |
| *ClKIN1A* | *Cla000622* | | CCCTACAGAAGCATTGCTAACCT | 139 |
|  |  |  | CCTCTTGTCCTTGGTTGATTCTT |  |
| *ClKIN1B* | *Cla011525* | | AGTTTCTTTGGAGACGGATTCTG | 199 |
|  |  |  | TTCCCCCAATCTTAGTAGTTCCA |  |
| *ClKIN1C* | *Cla013748* | | GTTAAGACGTTCATGCCAGTCAC | 211 |
|  |  |  | GTCATTCTCACTCACGGGTACAG |  |
| *ClKIN1D* | *Cla021298* | | GTGGCAATGAAAAACTACAGTCG | 114 |
|  |  |  | CTACTCCACGAGCAACTTGAGAA |  |
| *ClKIN4A* | *Cla023054* | | CCACAGAATTAAGACCCAAAAGG | 103 |
|  |  |  | CTGGAGAACTTCCTTCTCTCTCG |  |
| *ClKIN5A* | *Cla008272* | | ACGTACGGTTCTCGAGTTAAAGC | 209 |
|  |  |  | TATGCAGTCAGCTTCTCTTCCTG |  |
| *ClKIN5B* | *Cla017676* | | TGCTACAGTTTCTCCTGCTGTTC | 175 |
|  |  |  | AGCATAAACCTCTGCCTTCAAAC |  |
| *ClKIN5C* | *Cla018236* | | GAGATTGGATCATTCCCAAAGAC | 179 |
|  |  |  | CTACAGCATCAAAATCAGCATCC |  |
| *ClKIN7A* | *Cla001883* | | ACACATCAAGAATACGCCAGAGA | 122 |
|  |  |  | GGTCATCCAAGAGACGAAGAGAT |  |
| *ClKIN7B* | *Cla002884* | | GGCCACATACAATGAGAAAGAAG | 122 |
|  |  |  | TGACATCTCGTACAGCAGTCAGT |  |
| *ClKIN7C* | *Cla011614* | | AGAAGACGTTGGCTAGAATGGAC | 168 |
|  |  |  | CAGCAATGTAGCCTATGTGAAGG |  |
| *ClKIN7D* | *Cla013925* | | AAGAGAGAACACAAGCAGGTGAC | 175 |
|  |  |  | GCTGCTTGTTCTGACAGTCTTTT |  |
| *ClKIN7E* | *Cla014608* | | CTGCAAGGAAGTTCGATGTATTG | 144 |
|  |  |  | ACTATTTTCGAGGTGGTTGTGGT |  |
| *ClKIN7F* | *Cla015885* | | CAGAAGCGATTCTCAGAAGATGA | 216 |
|  |  |  | AATGAAGCTGAGACCAAAGTTCC |  |
| *ClKIN7G* | *Cla015893* | | CACACACTATGCATGGAGATCAA | 121 |
|  |  |  | TCAAGGTAAGATACACGGAGCAA |  |
| *ClKIN7H* | *Cla016598* | | AAGTACCGAAGGAAGAACCACTG | 200 |
|  |  |  | GGAACAAGCAAGAGAACAGGATT |  |
| *ClKIN7I* | *Cla018481* | | CGATTGTCAGCAGAAGAAAGAGA | 106 |
|  |  |  | GAGGATTTGTCCAGAGCTTGTTT |  |
| *ClKIN7J* | *Cla022444* | | TTCTAATGACCATCCCACCTTCT | 174 |
|  |  |  | CAGAATGTGAGTCTTTGGTGTCC |  |
| *ClKIN7K* | *Cla022645* | | CTGAGCGTATTGCTAAGACTGGA | 179 |
|  |  |  | ACCTAGAGCTGGTTGGAGTATGC |  |
| *ClKIN8A* | *Cla014076* | | AAGCAGGGTCTGACTATTGAAGG | 169 |
|  |  |  | GGACAAACATCATGCTGAGAAAC |  |
| *ClKIN8B* | *Cla019001* | | GACAGAGTTTGCTAATGGGAATG | 120 |
|  |  |  | GTCGTGGTGGAATAGACTTCTTG |  |
| *ClKIN10A* | *Cla003633* | | ACTACCATTATGCCAGAGGGTGT | 184 |
|  |  |  | AATTTGTGCTACTGGTCACCTCA |  |
| *ClKIN10B* | *Cla007599* | | CTGATGATTCTGCTTCTGCTGTT | 170 |
|  |  |  | CTCAAGCTTAGCTCTCAATGCTG |  |
| *ClKIN10C* | *Cla008965* | | TTCTTCCTTTAACTCCAGCAGTG | 184 |
|  |  |  | GACCTGGAATCTGTCAATCAAAG |  |
| *ClKIN10D* | *Cla011526* | | TAGCCCAACTGAAGAGATCAGAG | 220 |
|  |  |  | TGCACGAGAGAATCTAGAGAACC |  |
| *ClKIN10E* | *Cla017895* | | CTCTCTTCAACAGTTCTGCCAAA | 196 |
|  |  |  | AGTGGTGATAGAGCCTTTCTTGG |  |
| *ClKIN11A* | *Cla022414* | | GCTGATTCATCCATCGTTACAAG | 116 |
|  |  |  | CCCTCAGACTCCTTCACTTTGAT |  |
| *ClKIN12A* | *Cla004496* | | AAGAAGTCTATGTCTCGGCTTGG | 172 |
|  |  |  | TGTTATCCGGAGAGATAGCACAA |  |
| *ClKIN12B* | *Cla009212* | | CGTGAGGAGAAGATCAGACAACT | 217 |
|  |  |  | CACGCTCTCCTAGCTCATAAAAA |  |
| *ClKIN12C* | *Cla010272* | | ACAGCAGAGGTTACAACGAGAAG | 214 |
|  |  |  | CTCGGAGATATGGGCTTTAAGTT |  |
| *ClKIN12D* | *Cla018908* | | GGGCAGATGCTTCAAAGTCTTAT | 124 |
|  |  |  | CCCCTAAGTTCCATTTCAGATTG |  |
| *ClKIN12E* | *Cla021862* | | CTAAGTTTCAGCCATGTGCTTCT | 114 |
|  |  |  | GGGTATCGTAAACAGGCTCTTCT |  |
| *ClKIN13A* | *Cla009301* | | CTGGAAGCCACATTGAAAACTAC | 112 |
|  |  |  | TTAAGTCTATGCTGGAACCTTGC |  |
| *ClKIN13B* | *Cla010631* | | GGGTTGCAAGAATACAAAGTGTC | 175 |
|  |  |  | GAGCAGGCTTTGTTTCAGAACTA |  |
| *ClKIN13C* | *Cla015441* | | GCCATATTCCTTTTAGAGGCAGT | 153 |
|  |  |  | TCTTCACCCTGTCTGCATATCTT |  |
| *ClKIN14A* | *Cla001716* | | AGATCGACTGAAAGAAACCCAAG | 160 |
|  |  |  | CATCAACGTCTTTGAATCTCCAC |  |
| *ClKIN14B* | *Cla003337* | | CACATACAGTCTCAATGGAACGA | 209 |
|  |  |  | TAGTCCACCGTAGAATGCTGATT |  |
| *ClKIN14C* | *Cla005012* | | AGCAAAGGTAGTGGATTGTGTGT | 170 |
|  |  |  | AGCGTCTACAAGAGTCCAATGAG |  |
| *ClKIN14D* | *Cla005176* | | AGCCTTAGTGAAGAAGGATGGTG | 155 |
|  |  |  | CATCCTCCAGTGAGTTTGTTCTG |  |
| *ClKIN14E* | *Cla005413* | | GGAAACATTAGGGTCTTTTGTCG | 127 |
|  |  |  | GAGCCCCATTTGACTTAACAATC |  |
| *ClKIN14F* | *Cla008964* | | TGTCCAGTCTGCTGTAGATGGTT | 180 |
|  |  |  | GCCTTCAATGAGAACGAGAACTT |  |
| *ClKIN14G* | *Cla011288* | | AAAGATTCCTTAGGTGACGGTTC | 155 |
|  |  |  | TGCTTCTTCAAGTCCTCCTGTAG |  |
| *ClKIN14H* | *Cla011444* | | GGACCTGATGTATCATTGAGAGC | 102 |
|  |  |  | TTCATAGGAAATAGAGCCCTTCC |  |
| *ClKIN14I* | *Cla014106* | | CGGAATGCTGTATTAAGTCTTGG | 122 |
|  |  |  | CTGATTGCAGAGTGGAAGAAACT |  |
| *ClKIN14J* | *Cla017386* | | CTGCAATGTCCTGAATAAGGTTC | 181 |
|  |  |  | TAGATCAGACGCCTCAAAGGTAG |  |
| *ClKIN14K* | *Cla017457* | | CCGCTCTGAAGTTACAGGAGATA | 200 |
|  |  |  | CATCGGGATTAAGCTGTACAAAC |  |
| *ClKIN14L* | *Cla019422* | | TGTCTTCGGGAAGAACTACAACA | 114 |
|  |  |  | CAATGTTTCACCGTTGAACTCTC |  |
| *ClKIN14M* | *Cla019890* | | ATCTCGAGAGCCAACTAGCAGAT | 189 |
|  |  |  | TTCTTAGGGGAAGCCTTAGCTTT |  |
|  |  |  |  |  |
